# Elevated Creatine Kinase (CK) and Myositis Triggered by Immune Checkpoint Inhibitors: Muscle Mysteries Unveiled

**DOI:** 10.7759/cureus.71742

**Published:** 2024-10-17

**Authors:** Shivani K Modi, Ashish Subedi

**Affiliations:** 1 Internal Medicine, Einstein Medical Center Philadelphia, Philadelphia, USA; 2 Internal Medicine, Gandaki Medical College, Pokhara, NPL

**Keywords:** adverse events, immune checkpoint inhibitors, intravenous immunoglobulin, myositis, programmed death-ligand 1

## Abstract

Immune checkpoint inhibitors (ICIs), including programmed cell death protein 1 (PD-1) inhibitors, such as nivolumab, and cytotoxic T-lymphocyte-associated protein 4 (CTLA-4) inhibitors, such as ipilimumab, have revolutionized cancer treatment, particularly in metastatic melanoma. However, these therapies can cause immune-related adverse events (irAEs), including myositis, a rare but significant complication characterized by elevated creatine kinase (CK) and muscle weakness. We present the case of a 79-year-old male with a history of metastatic melanoma to the brain, previously treated with nivolumab and ipilimumab, who was admitted after an unwitnessed fall. Initial laboratory tests revealed significantly elevated CK levels (5277 U/L) and mildly elevated liver function tests (LFTs). Despite the absence of muscle pain or weakness upon presentation, the patient developed proximal muscle weakness and myalgias during hospitalization. Extensive workup, including negative autoimmune panels and imaging, raised suspicion of immune-related myositis given the recent ICI therapy. The patient was treated with prednisone, which resulted in a rapid decrease in CK levels and improvement of symptoms, supporting the diagnosis of ICI-related myositis.

## Introduction

Immune checkpoint inhibitors (ICIs) have emerged as a groundbreaking class of cancer therapeutics that harness the body's immune system to target and eliminate cancer cells. These agents work by blocking specific proteins on immune cells, such as programmed cell death protein 1 (PD-1), programmed death-ligand 1 (PD-L1), and cytotoxic T-lymphocyte-associated protein 4 (CTLA-4), which cancer cells exploit to evade immune detection. By inhibiting these checkpoint proteins, ICIs enhance the immune response against cancer cells, leading to improved survival outcomes in several malignancies, including melanoma, lung cancer, renal cell carcinoma, and more. Notable examples of these drugs include PD-1 inhibitors, such as nivolumab and pembrolizumab, and CTLA-4 inhibitors, such as ipilimumab [1].

Despite their substantial impact on cancer treatment, ICIs can trigger a spectrum of immune-related adverse events (irAEs) due to the non-specific activation of the immune system, which can attack normal tissues and organs. These irAEs are often inflammatory in nature and can affect virtually any organ system, ranging from mild dermatological reactions to severe and potentially life-threatening conditions. Common irAEs include dermatitis, colitis, hepatitis, pneumonitis, and endocrinopathies such as hypothyroidism and adrenal insufficiency [2]. While irAEs are generally manageable with immunosuppressive therapies such as corticosteroids, their presence poses a unique challenge in clinical oncology due to the need to balance cancer treatment efficacy with the management of these side effects.

Among the less frequently observed but clinically significant irAEs is myositis, an inflammatory muscle disease characterized by muscle weakness and elevated serum creatine kinase (CK) levels, reflecting muscle damage. ICI-induced myositis often manifests with proximal muscle weakness, myalgias, and, in some cases, respiratory and cardiac muscle involvement, which can complicate management [3]. The pathophysiology of ICI-related myositis is not fully understood but is believed to involve autoimmune-mediated muscle inflammation triggered by the enhanced immune response against shared antigens between cancer cells and normal muscle tissue.

Diagnosing myositis as an irAE can be challenging due to its non-specific symptoms, which may overlap with other common causes of muscle weakness, such as medication side effects, metabolic disorders, or other autoimmune conditions. The timing of symptom onset relative to ICI therapy, often within weeks to months after initiation, is a key diagnostic clue [4-6]. Comprehensive evaluation, including detailed clinical history, laboratory testing (e.g., CK levels, myositis-specific antibodies), imaging, and sometimes muscle biopsy, is essential for confirming the diagnosis and distinguishing ICI-related myositis from other etiologies.

Given the growing use of ICIs across multiple cancer types, recognizing and managing myositis as a potential irAE is crucial for oncologists and other healthcare providers. Prompt identification and appropriate immunosuppressive treatment, typically with corticosteroids, can lead to significant improvement in symptoms and reduce the risk of long-term muscle damage. However, the decision to continue or discontinue ICI therapy in the context of myositis must be individualized, weighing the benefits of cancer control against the severity of the autoimmune toxicity.

This study highlights a 79-year-old male with metastatic melanoma treated with nivolumab and ipilimumab who developed ICI-related myositis. The case underscores the importance of early recognition, multidisciplinary management, and the complexities involved in balancing oncologic treatment with the mitigation of irAEs.

## Case presentation

A 79-year-old male with a complex medical history, including deafness, hypertension (HTN), hyperlipidemia, epilepsy managed with levetiracetam (Keppra), chronic kidney disease (CKD) stage 3, and metastatic melanoma to the brain, presented to the emergency department (ED) following an unwitnessed fall. The patient denied any loss of consciousness during the fall and had no active complaints of muscle aches or weakness upon presentation. His cancer history included a left frontal craniotomy performed in January 2023 for metastatic melanoma.

In the ED, initial laboratory results were mostly unremarkable except for mildly elevated creatinine (Cr) at 1.47 mg/dL, troponin (Trop) at 0.12 ng/mL, elevated creatine kinase (CK) at 5277 U/L, and mildly elevated liver function tests (LFTs). A computed tomography angiogram (CTA) of the head and neck revealed postoperative changes related to the previous craniotomy, without acute findings. Electroencephalography (EEG) showed no active epileptiform activity. Intravenous fluids (IVF) were initiated as part of supportive management. An ultrasound of the right upper quadrant (RUQ USG) showed no acute abdominal abnormalities. Physical examination at this time was significant for decreased strength on the bilateral lower extremity (3/5). Everything else was normal (Table 1).

**Table 1:** Summarized laboratory results. CK: creatinine kinase; ANA: antinuclear antibodies; IFE: immunofixation electrophoresis; HMGCR: 3-hydroxy-3-methylglutaryl coenzyme-A; ALT: alanine aminotransferase

Test	Value	Reference range	Interpretation
CK	5277 U/L	30-135 U/L	Elevated, indicates muscle damage
Troponin	0.12 ng/mL	<0.01 ng/mL	Mildly elevated, no active ischemia
ANA by IFA	Negative	-	No autoimmune evidence
HMGCR antibody	Negative	-	No statin-related myositis
ALT	Elevated	7-56 U/L	Mild hepatotoxicity

During the hospital stay, the patient’s CK levels plateaued in the 3000s U/L. Approximately two days after admission, he began complaining of new-onset muscle weakness and aches in the proximal muscles of his arms and thighs. Notably, no skin rash was observed. Given these findings, a rheumatology consult was requested to evaluate for possible inflammatory myositis. The rheumatology team ordered several tests, including antinuclear antibody (ANA) testing by indirect immunofluorescence assay (IFA), a comprehensive myositis-specific antibody panel (myocyte panel 11 antibodies), and a 3-hydroxy-3-methylglutaryl-coenzyme A reductase (HMGCR) antibody test. Additionally, an MRI of the thighs was planned to identify a suitable site for muscle biopsy but it was not obtained over the weekend. Later, since the patient started having improvement on steroids, the rheumatologist did not want to perform an MRI of the thigh with a muscle biopsy, as it is invasive.

Upon further review of the patient’s medical history and prior treatment regimen, it was discovered that he had been receiving immune checkpoint inhibitor (ICI) therapy, specifically nivolumab (a PD-1 inhibitor) and ipilimumab (a CTLA-4 inhibitor), for his metastatic melanoma. His last dose of the ICI therapy was administered approximately 30 days before admission. The temporal association between the onset of his symptoms and recent ICI therapy raised a high suspicion for ICI-related myositis, an immune-related adverse event (irAE) associated with these therapies.

Given the clinical scenario, prednisone therapy was initiated at a dose of 1 mg/kg/day as the patient’s symptoms and elevated CK levels were suggestive of immune-mediated myositis. Following the initiation of corticosteroids, the patient showed significant improvement in muscle strength and a gradual reduction in CK levels, supporting the diagnosis of ICI-related myositis.

## Discussion

Immune checkpoint inhibitors (ICIs) such as nivolumab (a programmed cell death protein 1 {PD-1} inhibitor) and ipilimumab (a cytotoxic T-lymphocyte-associated protein 4 {CTLA-4} inhibitor) have significantly improved outcomes in various malignancies, including metastatic melanoma. However, the success of ICIs is tempered by the risk of immune-related adverse events (irAEs), which can affect multiple organ systems [5-8]. Myositis, a rare but increasingly recognized irAE, can present diagnostic and therapeutic challenges, particularly when clinical features are subtle or atypical, as demonstrated in this case [9].

ICI-related myositis is believed to result from the activation of autoreactive T cells, leading to an inflammatory response against muscle tissue. PD-1 and CTLA-4 pathways play crucial roles in maintaining immune tolerance, and their inhibition can trigger dysregulation of immune homeostasis, causing muscle inflammation. Although the exact mechanisms remain under investigation, ICIs can induce a spectrum of autoimmune phenomena, including myositis, myocarditis, and myasthenia gravis, often coexisting or overlapping. Elevated creatine kinase (CK) levels, muscle weakness, and myalgias are hallmark signs of myositis, though the severity and presentation can vary widely among patients [10-12].

Clinical features and diagnostic challenges

This case illustrates the complexity of diagnosing ICI-related myositis, especially when the clinical presentation lacks classic symptoms such as severe muscle pain or prominent weakness at initial presentation. Our patient, who had a recent history of ICI therapy, presented primarily with elevated CK levels and subtle muscle complaints that developed during hospitalization. Initially, there was no clear evidence of muscle inflammation, and the absence of overt rhabdomyolysis or cutaneous findings delayed the consideration of myositis.

The diagnosis of ICI-related myositis often requires a high index of suspicion, particularly in cancer patients on ICIs presenting with unexplained muscle weakness or elevated muscle enzymes [13]. Traditional diagnostic modalities, including MRI, electromyography, and muscle biopsy, can provide additional insights, though their utility may vary based on clinical context. In this case, the MRI of the thighs was planned to identify an appropriate biopsy site, but clinical judgment and timely recognition of the patient’s medication history were pivotal in raising suspicion for ICI-related myositis. This emphasizes the importance of integrating clinical history, physical examination, and targeted diagnostic testing to guide appropriate management.

Management of ICI-related myositis

Management of ICI-related myositis typically involves the discontinuation of the offending agent and initiation of immunosuppressive therapy, most commonly corticosteroids. Our patient responded well to prednisone therapy, with a notable reduction in CK levels and improvement in muscle strength, supporting the diagnosis of immune-mediated myositis. Corticosteroids are considered the first-line treatment, and early intervention is associated with better outcomes [14]. However, the dosing and duration of steroid therapy must be individualized, balancing the need to control the autoimmune process with the risk of steroid-related complications.

In cases of severe or refractory myositis, additional immunosuppressive agents such as methotrexate, mycophenolate mofetil, or intravenous immunoglobulin (IVIG) may be required. Furthermore, management may include close monitoring of other potential irAEs, as concurrent conditions such as myocarditis or myasthenia gravis can complicate the clinical course. Multidisciplinary collaboration, including input from oncology, rheumatology, and neurology, is often necessary to optimize patient care.

Challenges and considerations in the management of irAEs

The growing use of ICIs in cancer treatment necessitates an increased awareness of the broad spectrum of irAEs. Myositis represents a diagnostic challenge due to its variable presentation, and it requires careful clinical assessment, particularly in the absence of dramatic muscle symptoms. The overlap between ICI-related myositis and other irAEs, such as myocarditis and myasthenia gravis, further complicates the diagnosis, as these conditions can coexist in up to 25% of patients with ICI-induced muscle inflammation [15,16].

The timing of symptom onset is another critical factor, as irAEs can manifest at any point during or even after the completion of ICI therapy. In this case, the patient’s symptoms developed approximately one month after his last dose of nivolumab and ipilimumab, highlighting the need for continued vigilance for irAEs even in patients no longer actively receiving treatment.

The decision to restart ICI therapy after managing an irAE like myositis is complex and should be individualized. Factors such as the severity of the irAE, the patient’s cancer status, and alternative treatment options must be considered. In some cases, ICIs can be cautiously reintroduced with close monitoring, but the risk of recurrence of myositis and other irAEs remains a concern.

## Conclusions

This case underscores the critical importance of recognizing and managing ICI-related myositis, a potentially severe irAE that can significantly impact patient outcomes. Clinicians must maintain a high level of suspicion for irAEs in patients receiving ICIs, particularly when encountering unexplained symptoms such as muscle weakness and elevated CK levels. Early diagnosis and prompt initiation of immunosuppressive therapy are essential for achieving favorable outcomes. As the use of ICIs continues to expand across oncology, healthcare providers must be equipped with a comprehensive understanding of irAEs to ensure timely and effective management of these complex clinical scenarios.
